# Total Phenolic Content in Potato (*Solanum tuberosum* L.) Peel and Flesh Across Seed Generations

**DOI:** 10.3390/molecules31173014

**Published:** 2026-08-28

**Authors:** Rita Asakaviciute, Nijole Ruziene, Jolanta Jurkeviciute, Ingrida Radveikiene, Erika Kubiliene

**Affiliations:** Faculty of Agrotechnologies, Vilniaus Kolegija/Higher Education Institution, Studentu str. 39A, LT-08106 Vilnius, Lithuania; n.ruziene@atf.viko.lt (N.R.); j.jurkeviciute@atf.viko.lt (J.J.); i.radveikiene@atf.viko.lt (I.R.); e.kubiliene@viko.lt (E.K.)

**Keywords:** total phenolic content, Folin–Ciocalteu-reactive compounds, potato peel, potato flesh, seed propagation stage, *Solanum tuberosum* L.

## Abstract

This study compared total phenolic content (TPC) in potato tubers of four Lithuanian cultivars (*VB Venta*, *Goda*, *VB Meda*, and *VB Aista*) across three seed propagation stages. Folin–Ciocalteu-reactive compounds were analyzed in peel, flesh, and whole-tuber samples from three seed propagation stages: minitubers (m_1_), standard tubers (m_2_), and first-class certified seed potatoes (E_1_), using the Folin–Ciocalteu method. TPC was consistently highest in the peel, reaching 107.1 ± 5.3 µg gallic acid equivalents (GAE) g^−1^ dry weight (DW) in *VB Venta* (m_2_), whereas the highest flesh TPC (86.7 ± 4.3 µg GAE g^−1^ DW) was observed in *VB Meda* (E_1_). Across cultivars, peel TPC increased from 72.9 µg GAE g^−1^ DW in m_1_ to 85.6 µg GAE g^−1^ DW in E_1_. Whole-tuber TPC varied less among seed propagation stages (56.2–58.8 µg GAE g^−1^ DW). Because the seed propagation stages were produced under different cultivation conditions, the observed differences cannot be attributed solely to seed generation. The results confirm the higher concentration of Folin–Ciocalteu-reactive compounds in potato peel and indicate its potential for further investigation as a source of bioactive compounds.

## 1. Introduction

Potato (*Solanum tuberosum* L.) is one of the most important agricultural crops worldwide and ranks among the leading food crops in terms of global production, following wheat, rice, and maize [1]. Due to its major contribution to global food security, the United Nations declared 2008 the International Year of the Potato, highlighting its role in reducing hunger, poverty, and social exclusion [2]. Historically, the potato has played a transformative role in human development. Originating in the Andean region as a staple of the Inca civilization, it later became a key crop in Europe after its introduction in the sixteenth century, significantly contributing to population growth, urbanization, and agricultural stability [3]. In Lithuania, potatoes were first documented in 1629 and gradually became a staple crop, eventually earning the designation of the “second bread of Lithuanians” due to their central role in national diets and agriculture [4,5].

Beyond their historical and agronomic importance, potatoes are valued for their nutritional composition. They are a rich source of complex carbohydrates, dietary fiber, minerals (particularly potassium, magnesium, and iron), vitamins (notably vitamin C and B-group vitamins), and bioactive compounds such as phenolics [6,7]. Phenolic compounds are secondary metabolites involved in plant defense against biotic and abiotic stresses and are associated with antioxidant, anti-inflammatory, and antimicrobial properties beneficial to human health [8]. In potato tubers, phenolics are mainly represented by phenolic acids, including chlorogenic, caffeic, ferulic, and p-coumaric acids, as well as flavonoids such as quercetin, kaempferol, and anthocyanins, the latter being particularly abundant in colored genotypes [9]. Although the total phenolic content (TPC) in potatoes is generally lower than in fruits and berries, their high consumption makes them an important contributor to dietary antioxidant intake [10].

The accumulation and composition of phenolic compounds in potato tubers are influenced by multiple factors, including genotype, tuber color, environmental conditions, maturity, cultivation practices, storage, and processing methods. Considerable variation in TPC among potato cultivars has been reported, often exceeding a twofold difference [6]. In general, darker-colored tubers (purple and blue) tend to accumulate higher levels of phenolics and anthocyanins than light-colored varieties [9]. Importantly, phenolic compounds are unevenly distributed within the tuber, with the peel containing a substantially higher proportion—often up to 50% or more—of total phenolics compared with the flesh [11]. However, industrial peeling removes a significant share of these compounds, generating large quantities of peel waste that remain underutilized despite its potential as a source of natural antioxidants and functional ingredients [12]. Recent advances in extraction technologies, including ultrasound-assisted and supercritical fluid extraction, further highlight the potential of potato peel valorization within sustainable and circular bioeconomy frameworks [13].

Recent studies further support the concept that potato peel represents a valuable bioresource rather than a processing by-product. For example, the incorporation of potato peel powder into yogurt has been shown to improve physicochemical and sensory properties while exerting hypolipidemic effects in animal models [14]. In addition, potato peel-derived compounds, including proteins and bioactive peptides, have been recognized as promising ingredients for functional foods and nutraceutical applications [15]. Previous studies also demonstrated that the inclusion of potato peel extracts in dairy products can significantly improve lipid profiles by reducing cholesterol and triglyceride levels [14]. Collectively, these findings highlight the considerable potential of potato peel as a multifunctional raw material for food, feed, and biotechnological applications within sustainable bioeconomy systems. Therefore, the valorization of potato peel should be considered a key strategy within circular bioeconomy frameworks, enabling the simultaneous enhancement of nutritional quality, the reduction in processing waste, and the development of innovative food and feed products.

These findings indicate that potato peel may have potential as a value-added raw material; however, its practical utilization requires consideration of peel yield, extraction efficiency, processing requirements, and economic feasibility. Although previous studies have shown that phenolic content varies by genotype, tissue type (peel vs. flesh), and environmental conditions, limited information is available on how the seed propagation stage influences phenolic accumulation in potato tubers. This represents an important knowledge gap, particularly in the context of seed systems and quality-oriented production. Since total phenolic content is widely used as an indicator of antioxidant capacity and nutritional quality, a better understanding of its distribution across tuber tissues and seed propagation stages is essential.

Although tissue-specific differences in potato phenolic content, particularly between peel and flesh, have been extensively documented, considerably less information is available on how phenolic levels vary among successive seed propagation stages within the same cultivars. Such information may be relevant to understanding whether the biochemical variation observed in seed potato systems is associated with the propagation stage, cultivation environment, or their interaction.

Therefore, the aim of this study was to compare the total phenolic content in the peel, flesh, and whole tubers of four Lithuanian potato cultivars across three seed propagation stages (m_1_, m_2_, and E_1_). Because the seed propagation stages were produced under different cultivation conditions, the present study does not attempt to quantify an independent causal effect of seed generation. Rather, it provides a comparative assessment of propagation-stage-associated variation and identifies patterns that should be verified in future experiments conducted under standardized environmental conditions.

## 2. Results

### 2.1. Morphological and Culinary Characteristics of Lithuanian Potato Varieties

The main agronomic and culinary traits of four Lithuanian potato varieties—*VB Venta*, *Goda*, *VB Meda*, and *VB Aista*—are summarized in Table 1. These varieties differ in maturity class, skin and flesh color, cooking type, and taste score.

*VB Venta* is a very early cultivar registered in the EU in 2009, while *Goda* (registered in 2004) and *VB Meda* (registered in 2019) are early- and medium-maturing, respectively. *VB Aista*, registered in 2006, is a very late-maturing cultivar, representing the latest maturity group among those evaluated.

All cultivars have yellow skin, whereas flesh color varies from light yellow (*VB Venta*, *Goda*) to yellow (*VB Meda*) and white (*VB Aista*). Cooking types also differed: *VB Venta* was classified as type A (waxy), whereas *Goda*, *VB Meda*, and *VB Aista* belonged to type BC (medium–floury). Taste scores ranged from 7.2 to 8.6, with Goda achieving the highest score (8.6 ± 0.08) and *VB Aista* the lowest (7.2 ± 0.05).

As shown in Table 1, cultivars differed not only in agronomic and culinary traits but also in their total phenolic content, with *VB Venta* and *VB Meda* exhibiting the highest functional potential.

### 2.2. Varietal and Generational Differences in Phenolic Content

**Figure 1:**
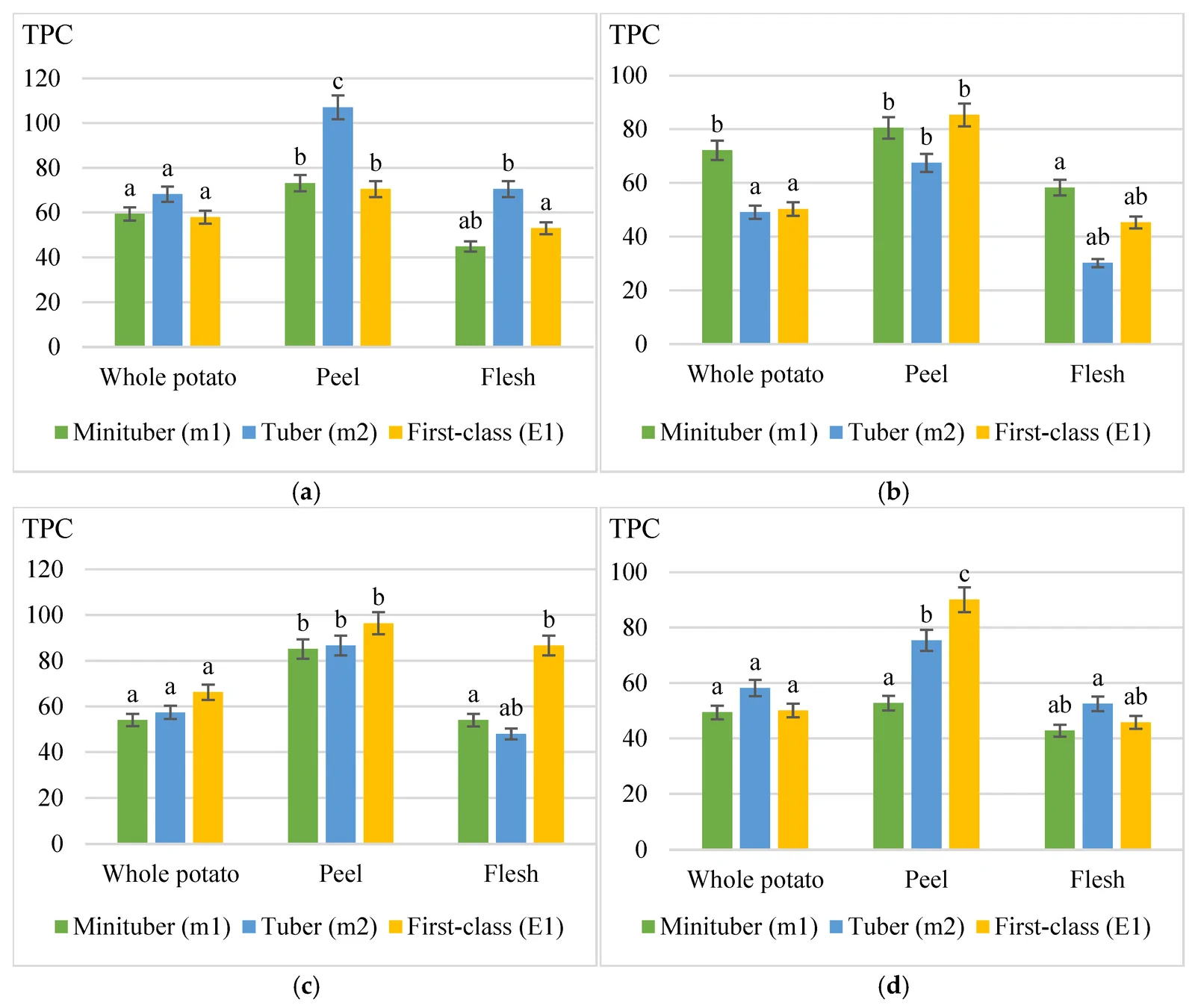
Total phenolic content (TPC, µg GAE g^−1^ DW) in whole-tuber, peel, and flesh samples of four potato cultivars across three seed propagation stages: (**a**) *VB Venta*, (**b**) *Goda*, (**c**) *VB Meda*, and (**d**) *VB Aista*. Values are presented as mean ± SE (*n* = 3). Different lowercase letters indicate significant differences according to Tukey’s HSD test (*p* < 0.01).

Significant variability in total phenolic content (TPC) was observed among cultivars, tuber tissues, and seed propagation stages (m_1_, m_2_, and E_1_) (Figure 1). Across all samples, the peel contained the highest phenolic concentrations, confirming that outer tissues are the main phenolic reservoir.

The highest peel phenolic content was recorded in *VB Venta* m_2_ tubers (107.1 ± 5.3 µg GAE g^−1^ DW). In this propagation stage, *VB Venta* also exhibited elevated phenolic levels in the flesh (59.6 ± 2.9 µg GAE g^−1^ DW) and whole tubers (68.2 ± 10.3 µg GAE g^−1^ DW). Conversely, *Goda* m_2_ tubers had the lowest phenolic content in the flesh (30.2 ± 1.5 µg GAE g^−1^ DW) and whole tubers (49.1 ± 6.3 µg GAE g^−1^ DW).

*VB Meda* demonstrated a distinctive pattern: the E_1_ propagation stage exhibited exceptionally high phenolic levels, particularly in the flesh (86.7 ± 4.3 µg GAE g^−1^ DW), which is uncommon given that inner tissues typically show much lower TPC values than the peel.

*VB Aista* displayed consistently lower phenolic content, especially in its m1 propagation stage (whole tubers: 49.4 ± 1.5 µg GAE g^−1^ DW; peel: 52.8 ± 2.6 µg GAE g^−1^ DW; flesh: 42.8 ± 2.1 µg GAE g^−1^ DW). However, peel phenolics markedly increased in the E_1_ propagation stage (90.1 ± 4.5 µg GAE g^−1^ DW).

### 2.3. Phenolic Distribution Among Seed Propagation Stages

**Figure 2:**
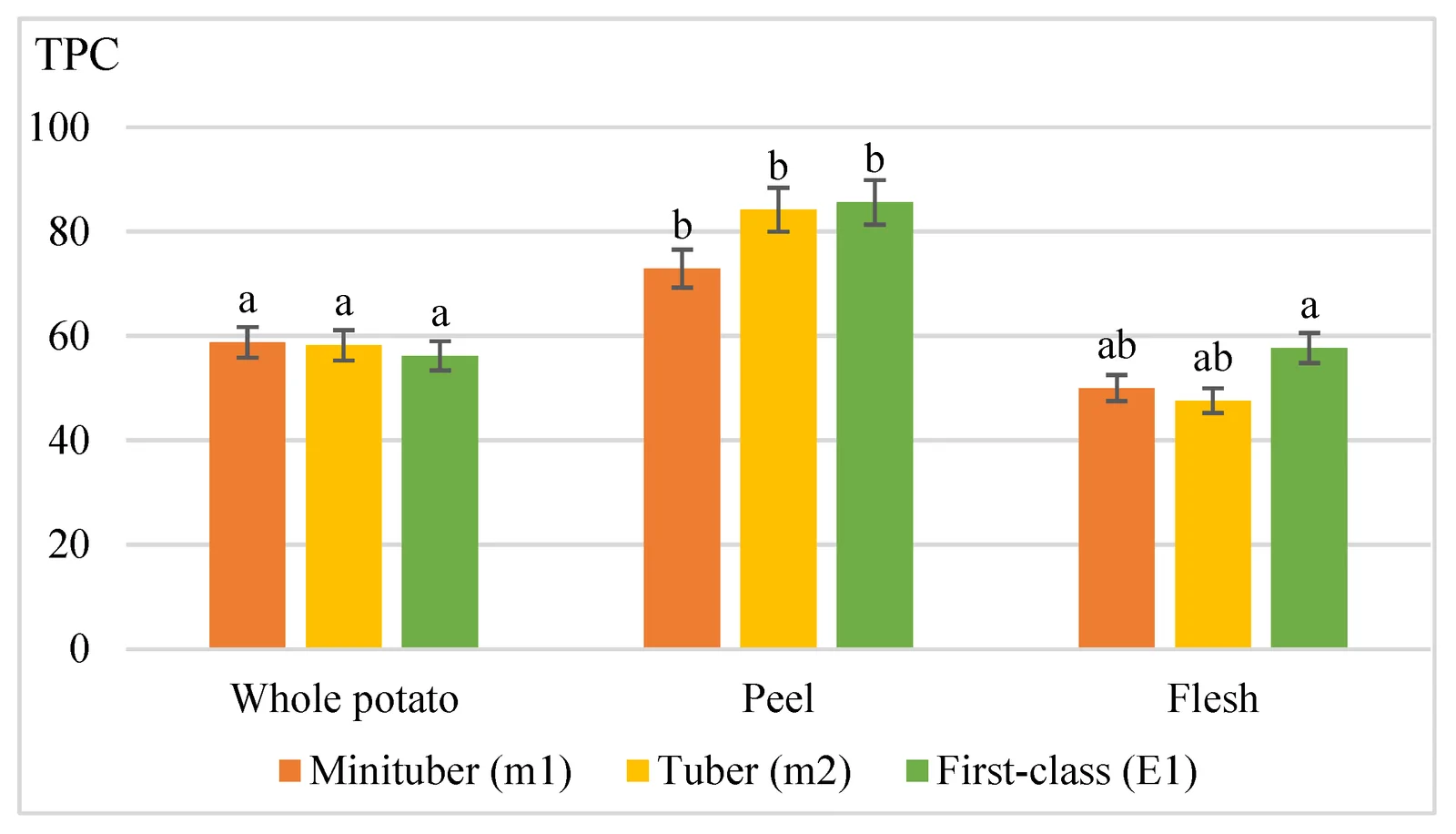
Total phenolic content (TPC, µg GAE g^−1^ DW) in ethanolic extracts of potato tubers across seed propagation stages. Values are presented as mean ± SE (*n* = 3). Different lowercase letters indicate significant differences according to Tukey’s HSD test (*p* < 0.01).

Independent of cultivar, peel phenolic content increased progressively from m1 to E_1_ seed material, ranging from 72.9 µg GAE g^−1^ DW in m1 to 85.6 µg GAE g^−1^ DW in E_1_ (Figure 2). A similar trend was observed in the flesh, where the highest total phenolic content was also recorded in E_1_ tubers (57.7 µg GAE g^−1^ DW), compared with m_1_ (50.0 µg GAE g^−1^ DW) and m_2_ (47.6 µg GAE g^−1^ DW). In contrast, phenolic content in whole tubers showed less variation among seed propagation stages, ranging from 56.2 to 58.8 µg GAE g^−1^ DW.

Overall, higher total phenolic content was observed in E_1_ material for some tissues and cultivars; however, because the seed propagation stages were produced under different cultivation conditions, these differences cannot be attributed solely to seed generation. This response was especially evident in the peel, which remained the main site of phenolic compound accumulation in potato tubers.

Based on the relative concentrations measured in the separated tissues, the peel represented approximately 50–61% of the combined peel-plus-flesh TPC index, while the flesh contributed 39–50%, depending on cultivar and seed propagation stage (Table 2). This confirms that the peel is the main site of phenolic accumulation and plays an important role in biochemical defense against environmental stresses and pathogens.

From a food quality perspective, these results indicate that peeling may remove a considerable proportion of bioactive compounds and reduce the nutritional value of potato products. However, the relatively high flesh contribution observed in some cases, particularly in *VB Meda* E_1_, suggests that both genotype and propagation stage can also influence internal phenolic accumulation. Therefore, peel-to-flesh variation is important both biologically and technologically, especially for peel valorization and minimally processed potato products.

These results demonstrate differences in phenolic accumulation among seed propagation stages and suggest that propagation-stage-associated and environmental factors may influence the biochemical characteristics of potato tubers.

## 3. Discussion

The present study revealed clear variation in total phenolic content (TPC) among potato cultivars, tuber tissues, and seed propagation stages. The most consistent pattern was the higher TPC observed in the peel compared with the flesh and whole-tuber samples. This distribution agrees with previous studies showing that potato periderm tissues generally contain higher concentrations of phenolic compounds than internal tissues, reflecting their role as a biochemical barrier exposed to environmental stresses and pathogen pressure [16,17,18].

An important limitation of the experimental design, however, is that the propagation stages were not produced under identical environmental conditions. Minitubers (m_1_) were obtained under controlled greenhouse conditions, whereas m_2_ and E_1_ tubers were produced under field conditions. Consequently, the observed differences cannot be attributed exclusively to the propagation stage. Environmental factors such as light exposure, temperature, water availability, soil conditions, and other cultivation-related variables may also have contributed to variation in the phenolic content. The results should therefore be interpreted as comparative differences among tubers representing distinct propagation stages and cultivation environments rather than as evidence of an independent causal effect of seed generation.

Differences were also observed among the four Lithuanian cultivars. *VB Venta* and *VB Meda* generally showed higher TPC values than *VB Aista*, although the magnitude of these differences depended on the tissue and propagation stage. Such cultivar-specific variation is consistent with previous reports demonstrating a strong genetic component in potato phenolic accumulation [6,19,20]. Genotype can influence both the overall concentration and tissue distribution of phenolic compounds, while environmental conditions further modify their accumulation.

The highest peel TPC was recorded in *VB Venta* m_2_ tubers (107.1 ± 5.3 µg GAE g^−1^ DW). However, because the m_2_ material was produced under field conditions, this relatively high value cannot be interpreted as a direct consequence of the propagation stage. Instead, it most likely represents the combined influence of cultivar, developmental status, and cultivation environment. Previous studies have shown that phenolic accumulation in potato tubers may vary in response to environmental and physiological conditions [20], but the present study did not investigate the biochemical mechanisms responsible for these differences.

A particularly notable result was the comparatively high TPC detected in the flesh of *VB Meda* E_1_ tubers (86.7 ± 4.3 µg GAE g^−1^ DW), approaching that measured in the peel. This pattern differed from the general trend toward substantially higher phenolic concentrations in outer tuber tissues. The result was reproducible among analytical replicates; nevertheless, its biological origin cannot be established from the present experiment. The Folin–Ciocalteu assay measures overall reducing capacity and is not strictly specific to phenolic compounds. Potato tubers also contain ascorbic acid, reducing sugars, and other reducing substances that may contribute to the measured response. Therefore, possible matrix-related interference cannot be excluded. Confirmation using chromatographic methods such as HPLC-DAD or LC-MS would be necessary to determine whether the elevated response in *VB Meda* E_1_ flesh reflects higher concentrations of individual phenolic compounds.

The distribution of phenolic compounds between the peel and flesh further demonstrated marked tissue-specific differences. Based on the relative concentrations measured in the separated tissues, peel represented approximately 50–61% of the combined peel-plus-flesh TPC index, depending on cultivar and propagation stage. However, these values should not be interpreted as the actual proportion of total tuber phenolics located in the peel because peel and flesh mass fractions were not determined. A true mass-balance assessment would require the measurement of peel yield relative to whole-tuber mass together with tissue-specific phenolic concentrations.

From a food-quality perspective, the consistently higher concentration of phenolic compounds in potato peel indicates that this fraction warrants further investigation as a potential source of bioactive constituents. Potato peel is already recognized as a promising by-product for the recovery of phenolic compounds and other potentially valuable components [12,13,21,22]. Nevertheless, the present study assessed neither peel yield nor extraction recovery, processing suitability, safety, or economic feasibility. Therefore, the results support the biochemical potential of potato peel but do not by themselves demonstrate its suitability for industrial valorization or functional food applications.

The differences observed among propagation stages were not uniform across cultivars or tissues. For example, E_1_ material showed relatively high TPC in the peel and flesh when averaged across cultivars, whereas whole-tuber values varied less among propagation stages. These patterns suggest that the biochemical composition differs among tubers produced at different propagation stages and under different cultivation conditions. However, physiological aging, environmental exposure, tuber maturity, and storage history may all contribute to this variation and were not independently controlled in the present experiment.

Tuber physiological maturity and detailed postharvest storage conditions were not systematically recorded for all propagation stages. Both factors may influence phenolic metabolism and therefore represent additional sources of variation. Similarly, potential mechanisms involving phenylpropanoid metabolism, oxidative stress, hormonal status, or enzyme activities were not investigated. Accordingly, explanations involving physiological aging, phenylalanine ammonia-lyase activity, polyphenol oxidase activity, or stress signaling should be regarded as hypotheses requiring experimental verification rather than as mechanisms demonstrated by the present study.

The Folin–Ciocalteu assay itself represents another important methodological limitation. Although it is widely used for the comparative estimation of total phenolic content, the reaction is not strictly specific to phenolic compounds and may also detect other reducing substances. Consequently, the values reported here are more appropriately interpreted as comparative estimates of Folin–Ciocalteu-reactive compounds. Future studies should complement this assay with chromatographic profiling to identify and quantify individual phenolic acids and flavonoids, particularly chlorogenic acid and related compounds that are characteristic of potato tubers [9,20,23].

Finally, TPC represents only one component of potato biochemical quality. Major nutritional and technological constituents such as starch, soluble carbohydrates, reducing sugars, and ascorbic acid were not quantified. Therefore, the present results should not be interpreted as a comprehensive assessment of nutritional quality. Future experiments should integrate phenolic profiling with carbohydrate composition, antioxidant activity, physiological maturity, storage parameters, and agronomic characteristics. Importantly, different propagation stages should be cultivated under comparable environmental conditions, preferably across multiple seasons or locations, to separate propagation-stage effects from environmental influences.

Overall, the present study demonstrates substantial cultivar- and tissue-associated variation in phenolic content in Lithuanian potato germplasm. The consistently higher concentrations observed in the peel support further investigation of this tissue as a potential source of bioactive compounds, whereas the unusually high response detected in the *VB Meda* E_1_ flesh warrants confirmation using compound-specific analytical methods. The results provide a basis for more controlled studies designed to distinguish among genetic, environmental, physiological, and propagation-related contributions to potato phenolic composition.

## 4. Materials and Methods

### 4.1. Plant Material and Experimental Sites

**Figure 3:**
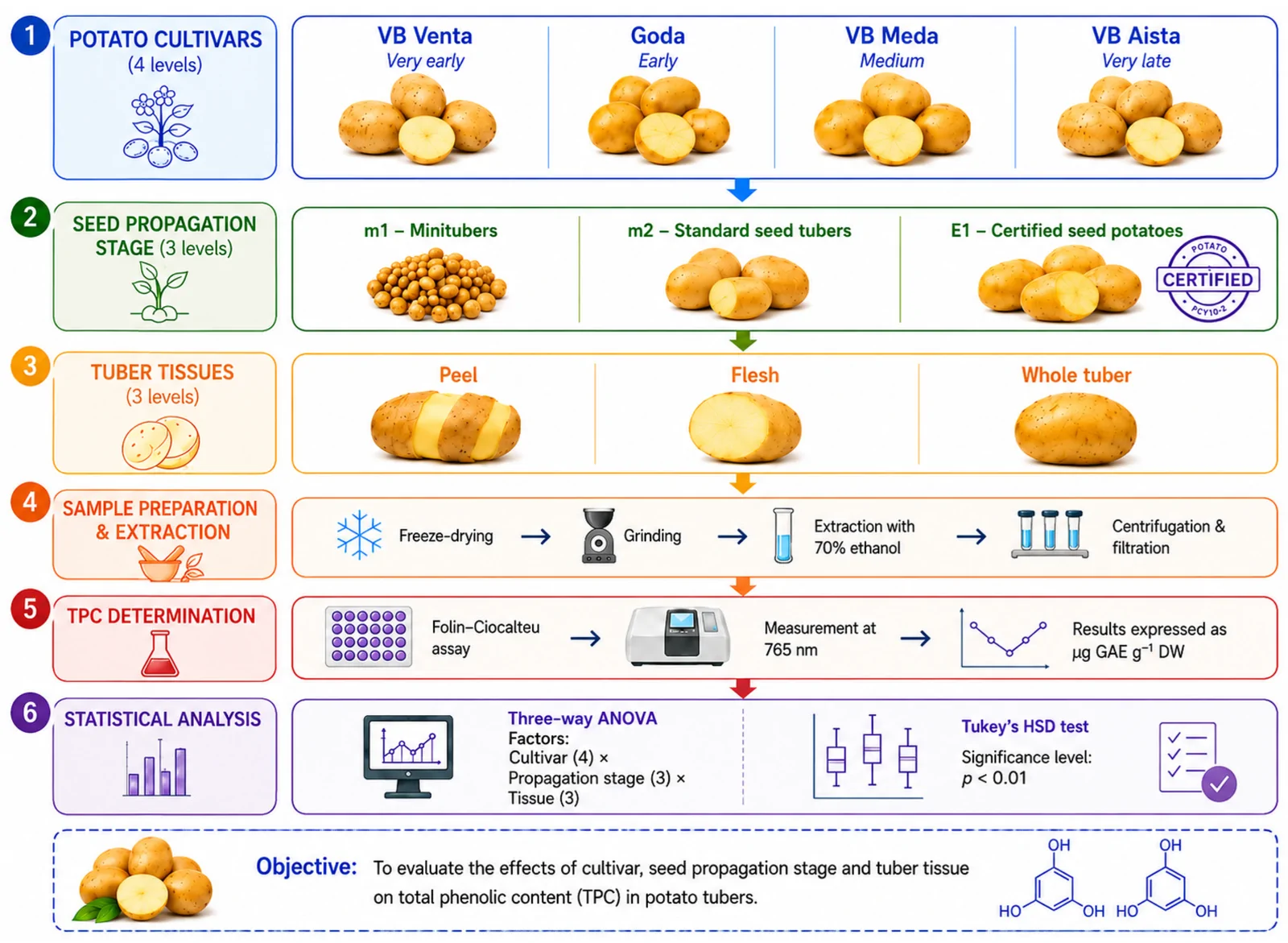
Schematic representation of the experimental design used to determine the total phenolic content (TPC) in potato tubers. The figure was created by the authors with the assistance of ChatGPT-5 (OpenAI, San Francisco, CA, USA) and subsequently reviewed and edited by the authors for scientific accuracy.

The study evaluated the total phenolic content (TPC) in potato tubers of four Lithuanian cultivars: *VB Venta* (very early), *Goda* (early), *VB Meda* (medium), and *VB Aista* (very late). All potato plant material used in this study was obtained from the Vokė Branch of the Institute of Agriculture, Lithuanian Research Centre for Agriculture and Forestry (Vilnius, Lithuania). The experiment followed a factorial structure including three factors: cultivar (4 levels), seed propagation stage (3 levels), and tuber tissue (3 levels). The seed propagation stages consisted of minitubers (m_1_), standard tubers (m_2_), and first-class certified seed potatoes (E_1_). Phenolic compounds were analysed separately in the peel, flesh, and whole tubers (Figure 3).

An important limitation of the present study is that total phenolic content was determined using the Folin–Ciocalteu assay, which is based on overall reducing capacity and is not strictly specific to phenolic compounds. Therefore, the values reported in this study should be interpreted as comparative estimates of total phenolic content rather than as a detailed characterization of the phenolic composition. Further studies using HPLC-DAD or LC-MS are required to identify and quantify individual phenolic compounds, particularly chlorogenic acid and other major phenolic acids, and to determine their contribution to the differences observed among potato cultivars, tuber tissues, and seed propagation stages.

### 4.2. Environmental Conditions and Crop Management

Minitubers (m_1_) were produced under controlled greenhouse conditions at the Vokė Branch of the Lithuanian Research Centre for Agriculture and Forestry. Plants were grown at 18 °C during a 12 h photoperiod and 12 °C during a 12 h dark period, with 85% relative humidity, supplemental lighting, and regular irrigation.

Standard tubers (m_2_) and certified seed tubers (E_1_) were produced under field conditions at the same experimental site. The soil type was sandy loam *Haplic Luvisol*, characterised by pH_k_Cl 5.1–5.5, humus content ≤ 2.0%, available P_2_O_5_ 180–240 mg kg^−1^, and K_2_O 150–190 mg kg^−1^. Each experimental plot covered 4.9 m^2^ and consisted of two rows with 20 tubers per plot. Fertilization was applied at N_90_P_90_K_90_, and standard agronomic practices such as harrowing, hilling, and plant protection were performed according to local cultivation recommendations. Field-grown tubers were produced under the standard environmental conditions of the Vokė experimental site, and the main soil and agronomic parameters are provided to ensure reproducibility.

Tuber physiological maturity and detailed postharvest storage conditions were not systematically recorded for all seed propagation stages and were therefore not included as controlled experimental factors.

### 4.3. Chemicals

Sodium carbonate (anhydrous, ACS reagent, ≥99.5%), gallic acid (97.5–102.5%, titration), and Folin–Ciocalteu phenol reagent were obtained from Sigma-Aldrich (Buchs, Switzerland). Food-grade ethanol was obtained from Vilniaus Degtinė (Vilnius, Lithuania). A 70% (*v*/*v*) aqueous ethanol solution was used for the extraction of phenolic compounds.

### 4.4. Extraction of Phenolic Compounds

For phenolic analysis, tubers were washed and manually separated into peel and flesh fractions, while whole tubers were analyzed as a combined sample. The material was homogenized prior to extraction. Free phenolic compounds were extracted following a modified protocol based on Zhou et al. [24]. The samples were dried at 60 ± 2 °C for 6–8 h in a DO55 drying oven (DragLab Technologies GmbH, Eschborn, Germany). One gram of dried plant material was mixed with 10 mL of 70% (*v*/*v*) ethanol and homogenised for 3 min at 25 Hz and 20 °C using an MM400 Mixer Mill (Retsch GmbH, Haan, Germany). The homogenate was centrifuged at 4500× *g* for 5 min using a BKC-TH 16II centrifuge (Biobase, Jinan, China). The supernatant was collected for subsequent analyses.

### 4.5. Determination of Total Phenolic Content (TPC)

TPC was quantified using the Folin–Ciocalteu spectrophotometric method [25]. One milliliter of extract was mixed with 5 mL of 10-fold diluted Folin–Ciocalteu reagent and incubated for 10 min at room temperature. Subsequently, 4 mL of 7.5% sodium carbonate solution was added, followed by a 30 min incubation in the dark. Absorbance was measured at 765 nm using a UV/Vis spectrophotometer (UV5, Mettler Toledo, Greifensee, Switzerland) with 10 mm quartz cuvettes (Hellma, Müllheim, Germany).

The relative contribution of peel and flesh to total phenolic content was calculated as a percentage of their combined TPC values within each sample. Results were expressed as micrograms of gallic acid equivalents per gram of dry weight (µg GAE g^−1^ DW), based on a calibration curve prepared using gallic acid standards (R^2^ = 0.999).

No specific correction for reducing sugars, ascorbic acid, or other non-phenolic reducing substances was applied, and chromatographic confirmation was not performed. Therefore, the Folin–Ciocalteu results should be interpreted as comparative estimates of Folin–Ciocalteu-reactive compounds rather than as a specific measure of total phenolic compounds.

### 4.6. Statistical Data Analysis

Statistical analyses were performed using IBM SPSS Statistics, version 20.0 (IBM Corp., Armonk, NY, USA). The effects of cultivar, propagation stage, and tuber tissue on total phenolic content (TPC) were evaluated using a three-way analysis of variance (ANOVA) according to a 4 × 3 × 3 factorial design. The fixed factors were cultivar (*VB Venta*, *Goda*, *VB Meda*, and *VB Aista*), propagation stage (m_1_, m_2_, and E_1_), and tissue type (peel, flesh, and whole tuber). The main effects of each factor and their two-way and three-way interactions (cultivar × propagation stage, cultivar × tissue, propagation stage × tissue, and cultivar × propagation stage × tissue) were assessed. When significant differences were detected, means were compared using Tukey’s honestly significant difference (HSD) post hoc test. Differences were considered statistically significant at *p* < 0.01. Data are presented as the mean ± standard error (SE).

## 5. Conclusions

In conclusion, potato peel generally contained higher levels of phenolic compounds than the flesh; however, the magnitude of this difference varied among cultivars and seed propagation stages. In particular, *VB Meda* E_1_ showed a comparatively high phenolic content in the flesh, approaching that of the peel.

From an applied perspective, the higher concentration of phenolic compounds observed in potato peel indicates that this fraction warrants further investigation as a potential source of bioactive compounds. However, the present study did not assess peel yield, extraction efficiency, processing suitability, safety, or economic feasibility; therefore, its practical valorization requires additional dedicated studies.

Future research should focus on (i) advanced characterization of phenolic profiles using chromatographic techniques such as HPLC or LC–MS, (ii) evaluation of antioxidant and biological activities in vitro and in vivo, and (iii) investigation of the effects of genotype, environmental conditions, physiological maturity, and storage on phenolic accumulation. Further studies should also assess peel yield, recovery efficiency, processing feasibility, and techno-economic aspects before conclusions regarding industrial or functional food applications can be drawn.

## Figures and Tables

**Table 1:** Agronomic, culinary, and functional characteristics of Lithuanian potato cultivars in relation to total phenolic content (TPC).

Characters	Potato Varieties			
	*VB Venta*	*Goda*	*VB Meda*	*VB Aista*
Year of EU registration	2009	2004	2019	2006
Maturity class	Very early	Early	Medium	Very late
Parentage	*Priekuliu visagrie* × *Pirmūnės*	*Ausonia* × *Franzi*	*Matilda* × No.3093	No.263 × No.476-9
Skin color	Yellow	Yellow	Yellow	Yellow
Flesh color	Light yellow	Light yellow	Yellow	White
Cooking type	A	BC	BC	BC
Taste score (points)	8.0 ± 0.09 ^b^	8.6 ± 0.08 ^a^	8.2 ± 0.07 ^b^	7.2 ± 0.05 ^c^
Mean TPC in peel (µg GAE g^−1^ DW)	95.3 ± 6.1 ^a^	84.6 ± 5.2 ^ab^	88.9 ± 6.4 ^ab^	71.0 ± 4.8 ^b^
Mean TPC in flesh (µg GAE g^−1^ DW)	55.8 ± 3.1 ^ab^	40.5 ± 2.2 ^c^	63.4 ± 5.0 ^a^	46.3 ± 2.7 ^bc^
Mean TPC in whole tuber (µg GAE g^−1^ DW)	61.5 ± 6.1 ^a^	52.3 ± 4.7 ^a^	58.9 ± 5.9 ^a^	49.6 ± 3.1 ^a^
Peel contribution to total TPC (%)	59.2 ^a^	57.9 ^ab^	52.6 ^b^	57.6 ^ab^

Note: cooking type: A—firm (waxy); B—fairly firm; C—floury; TPC values represent means across seed propagation stages (m_1_, m_2_, and E_1_) and are expressed as mean ± standard error (SE), with *n* = 3. Within each row, means followed by different lowercase superscript letters indicate significant differences among cultivars (*p* < 0.01).

**Table 2:** Relative contribution (%) of peel and flesh to total phenolic content (TPC) in potato tubers across seed propagation stages (values represent means ± SE; *n* = 3).

Variety	Seed Generation	Peel Contribution (%)	Flesh Contribution (%)	Peel/Flesh Ratio
*VB Venta*	m_1_	57.2 ± 3.62	42.8 ± 2.01	1.34
	m_2_	61.1 ± 5.32	38.9 ± 2.90	1.57
	E_1_	59.3 ± 3.51	40.7 ± 2.64	1.46
*Goda*	m_1_	54.8 ± 4.07	45.2 ± 1.53	1.21
	m_2_	60.3 ± 3.36	39.7 ± 1.52	1.52
	E_1_	58.6 ± 4.20	41.4 ± 2.28	1.41
*VB Meda*	m_1_	52.1 ± 4.25	47.9 ± 2.61	1.09
	m_2_	55.4 ± 4.30	44.6 ± 2.39	1.24
	E_1_	50.3 ± 4.86	49.7 ± 4.33	1.01
*VB Aista*	m_1_	55.2 ± 2.62	44.8 ± 2.19	1.23
	m_2_	57.6 ± 3.74	42.4 ± 2.60	1.36
	E_1_	60.1 ± 4.54	39.9 ± 2.28	1.51

Note: values represent the mean ± standard error (SE) (*n* = 3). The peel-to-flesh ratio was calculated solely as a descriptive indicator of the relative distribution of phenolic compounds between tuber tissues and should not be interpreted independently of the corresponding absolute TPC values.

## Data Availability

The original contributions presented in this study are included in the article. Further inquiries can be directed to the corresponding author.
